# The Australian living guidelines for the clinical care of people with COVID-19: What worked, what didn’t and why, a mixed methods process evaluation

**DOI:** 10.1371/journal.pone.0261479

**Published:** 2022-01-07

**Authors:** Tari Turner, Julian Elliott, Britta Tendal, Joshua P. Vogel, Sarah Norris, Rhiannon Tate, Sally Green, on behalf of the National COVID-19 Clinical Evidence Taskforce

**Affiliations:** 1 School of Public Health and Preventive Medicine, Monash University, Melbourne, Australia; 2 Infectious Diseases Unit, Alfred Health, Melbourne, Australia; 3 Maternal, Child and Adolescent Health Program, Burnet Institute, Melbourne, Australia; 4 School of Public Health, University of Sydney, Sydney, Australia; Deakin University, AUSTRALIA

## Abstract

**Introduction:**

The Australian National COVID-19 Clinical Evidence Taskforce is producing living, evidence-based, national guidelines for treatment of people with COVID-19 which are updated each week. To continually improve the process and outputs of the Taskforce, and inform future living guideline development, we undertook a concurrent process evaluation examining Taskforce activities and experience of team members and stakeholders during the first 5 months of the project.

**Methods:**

The mixed-methods process evaluation consisted of activity and progress audits, an online survey of all Taskforce participants; and semi-structured interviews with key contributors. Data were collected through five, prospective 4-weekly timepoints (beginning first week of May 2020) and three, fortnightly retrospective timepoints (March 23, April 6 and 20). We collected and analysed quantitative and qualitative data.

**Results:**

An updated version of the guidelines was successfully published every week during the process evaluation. The Taskforce formed in March 2020, with a nominal start date of March 23. The first version of the guideline was published two weeks later and included 10 recommendations. By August 24, in the final round of the process evaluation, the team of 11 staff, working with seven guideline panels and over 200 health decision-makers, had developed 66 recommendations addressing 58 topics. The Taskforce website had received over 200,000 page views. Satisfaction with the work of the Taskforce remained very high (>90% extremely or somewhat satisfied) throughout. Several key strengths, challenges and methods questions for the work of the Taskforce were identified.

**Conclusions:**

In just over 5 months of activity, the National COVID-19 Clinical Evidence Taskforce published 20 weekly updates to the evidence-based national treatment guidelines for COVID-19. This process evaluation identified several factors that enabled this achievement (e.g. an extant skill base in evidence review and convening), along with challenges that needed to be overcome (e.g. managing workloads, structure and governance) and methods questions (pace of updating, and thresholds for inclusion of evidence) which may be useful considerations for other living guidelines projects. An impact evaluation is also being conducted separately to examine awareness, acceptance and use of the guidelines.

## Introduction

Living guidelines are a new approach to developing and maintaining rigorous evidence-based guidelines in which any new evidence is rapidly incorporated, ensuring that recommendations are continually up to date with the latest research [1]. Living guideline methods are particularly useful in clinical areas in which research and practice are rapidly developing, of which COVID-19 is a clear example.

A small but increasing number of living guideline projects are underway, including in stroke, diabetes, maternal health and the recent living WHO guideline on drugs for COVID-19 [2–5], however little is currently known about what characteristics of the design or conduct of these projects make them more or less likely to succeed.

The Australian National COVID-19 Clinical Evidence Taskforce is a consortium of 32 Australian health professional organisations representing the full range of health professionals providing care to Australians with COVID-19, co-funded by Australian national and state governments and philanthropic organisations. The Taskforce is developing living guidelines for care of people with suspected or confirmed COVID-19 which are updated each week to reflect new evidence. The structure and methods used by the Taskforce to develop the guidelines have been described previously [6]. In brief, the guidelines use the rigorous GRADE (Grading of Recommendations Assessment, Development and Evaluation) approach [7] and are designed to meet Australian National Health and Medical Research Council (NHMRC) standards [8]. Each week, the team identify and review new evidence; convene multiple multidisciplinary guideline panels; revise existing recommendations and make new recommendations. The guideline recommendations are published and freely accessible online in the MAGICapp online guideline platform, and are disseminated widely through mainstream and social media, and promoted widely by Taskforce member organisations [9].

Given the novel approach, unique opportunity to study rapid and living guideline development in new disease and the need to continuously improve processes in this rapidly evolving guideline, we undertook a process evaluation exploring the activity and experience of participants in the Taskforce for the first 5 months of the living guidelines project. The aim of the process evaluation was two-fold: to enable us to improve process and outputs of the Taskforce as guideline development was underway, and to identify factors that might be useful to inform design and development of future living guidelines, by capturing the activities and experiences of Taskforce contributors each month during the living guidelines project.

## Methods

This process evaluation utilised a mixed methods approach consisting of activity and progress audits, online surveys, and semi-structured interviews. A protocol was developed for the process evaluation by TT. The protocol was refined in discussion with the Taskforce Executive team and approved by the Taskforce Steering Committee. Ethics approval was provided by Monash University Human Research Ethics Committee (Project ID: 24536).

Data were collected through five, prospective 4-weekly timepoints (beginning first week of May) and three, fortnightly retrospective timepoints (March 23, April 6 and 20). At each 4-weekly prospective timepoint we conducted an online survey of all participants in the Taskforce, as well as semi-structured interviews with key contributors and an audit of activity and progress. We collected and analysed both quantitative and qualitative data, taking a pragmatic approach focusing on rapidly informing Taskforce decision-making. For retrospective timepoints, we collected and analysed quantitative activity data only. The data underlying the results presented in this study are available on the Monash University Data Repository–BRIDGES (https://bridges.monash.edu/articles/dataset/National_COVID19_Clinical_Evidence_Taskforce_Process_Evaluation_Data/16926868).

### Activity audit

Every 4 weeks, data were collected on a set of markers of the activity and progress of the Taskforce, outlined in Box 1. Data were collected from administrative reports (including Google Analytics), individual team members and MAGIC.

**Box 1. Data collected in the activity audit**

Number of recommendations made in guidelineNumber of clinical topics addressed by guideline

Number of citations screened by Evidence Team

Number of references included in guideline

Number of guideline panels convened

Number of guideline panel meetings conducted

Number of Slack messages sent by Evidence Team

Number of staff employed

Number of individuals engaged in Taskforce panels, committees, groups, etc

Number of Taskforce member organisations

Number of representative members in the Jurisdictional Liaison Group

Number of Taskforce funders

Number of Taskforce partners

Number of views of the Taskforce webpage Proportion of Taskforce webpage views from Australia

Number of Taskforce media mentions

Number Australian COVID-19 cases

Number of Australian COVID-19 deaths

Number of COVID-19 trials on ClinicalTrials.gov

### Online survey

All members of Taskforce Executive, Steering Committee, Guideline Leadership Group, Guideline and Consumer Panels and the Evidence Team were invited to complete an online survey via direct email, sent every four weeks. This included both paid staff and voluntary contributors to the Taskforce (mostly clinicians). The survey was open for a week in each cycle. Participation was anonymous and voluntary, and completion of the online survey after reading the explanatory statement was considered implied consent.

Both quantitative and qualitative data were collected using an online survey tool (Qualtrix). Participants were asked to describe:

- Overall level of satisfaction with the work of the National COVID-19 Clinical Evidence Taskforce (5-point Likert: Extremely satisfied, Somewhat satisfied, Neither satisfied nor dissatisfied, Somewhat dissatisfied, Extremely dissatisfied)
- What is working well? (Free text)
- What could be improved? (Free text)
- With the benefit of hindsight, what would you do differently next time? (Free text)

Quantitative data was analysed using simple descriptive statistics, qualitative (free text) data were analysed in combination with the interview data.

### Interviews

Interview participants included the Taskforce Executive Team, and purposively selected individuals with other roles in the Taskforce including clinical content experts and members of the evidence synthesis team. Taskforce members have a diverse range of roles. The Executive Team is comprised of clinicians, methodology experts, researchers, and the Taskforce communications and engagement manager, evidence and methodology manager, business manager and administration staff. Evidence team members largely have health science backgrounds and experience in evidence synthesis methodologies. Participants were invited to participate via direct email which included the explanatory statement. Participation was voluntary and agreeing to conduct the interview was considered consent. Five to ten semi-structured interviews were conducted with key members of the Executive Team and different Taskforce members during each of the five, four-weekly data collection cycles. Interviews were conducted via Zoom and were audio-recorded. Detailed notes were also taken. Interview questions were based on a predetermined interview schedule, with questions varied to suit the interviewee’s roles and experience. The interview schedule was developed by one author (TT) in consultation with the other authors.

Data were collected on:

- Satisfaction with progress, what was working well, which areas needed improvement
- Challenges, barriers, facilitators and enablers encountered
- What they might do differently with the benefit of hindsight
- Expectations for the next month
- What one thing would make the greatest difference to the work of the Taskforce

Interviews were conducted by one author (TT) who has extensive experience in qualitative interviewing, and data were de-identified. TT was known to the interviewees, some of whom are long-standing colleagues, and some of whom have been introduced during the work of the Taskforce. None of the participants were managed by or in a reporting relationship with TT. Each month, the interview data were combined with qualitative data from the survey, and were analysed using open coding to identify key concepts which were organised into emerging themes. TT undertook the primary data analysis, SG collaborated on the conceptual development and refining of themes. For this analysis, we primarily reviewed the reports to identify themes across time, but also revisited the primary data as necessary to confirm interpretation and review coding.

### Reporting

Every 4 weeks, results from the activity audit, survey and interviews were analysed and a brief report prepared for the Taskforce Steering Committee. Data were also used contemporaneously by the Taskforce Executive to refine Taskforce processes and outputs.

## Results

### Activity audits

The Taskforce formed in late March 2020, with a nominal start date of March 23. The first version of the guideline was published two weeks later and included 10, initially primarily consensus, recommendations. Using the rigorous, evidence-based methods described in detail elsewhere [6], the guidelines have been revised, updated and republished each week since.

Levels of activity were very high in the first month of Taskforce. The focus was on securing funding, rapidly establishing the team, convening three guideline panels covering mild, moderate-severe and critical COVID-19, engaging 20 partner organisations and establishing fundamental governance structures.

In May and June, the Taskforce built on this initial base to establish clear operational workflows and processes, expanded to include six guideline panels (addressing questions relevant to pregnant women, children, adolescents, older people, and people requiring palliative care); further developed engagement with jurisdictional decision makers (Commonwealth and state government groups) and strengthened the communications function.

By August 24, in the final round of the process evaluation, five months after establishment, the team consisted of 11 full-time equivalent staff, working with seven guideline panels and over 200 contributing individuals (health policymakers, practitioners and other stakeholders).

The evidence identified had informed development of 66 recommendations (version 18). The Taskforce website had received over 200,000 total pageviews and 116,383 unique visits.

**Fig 1:**
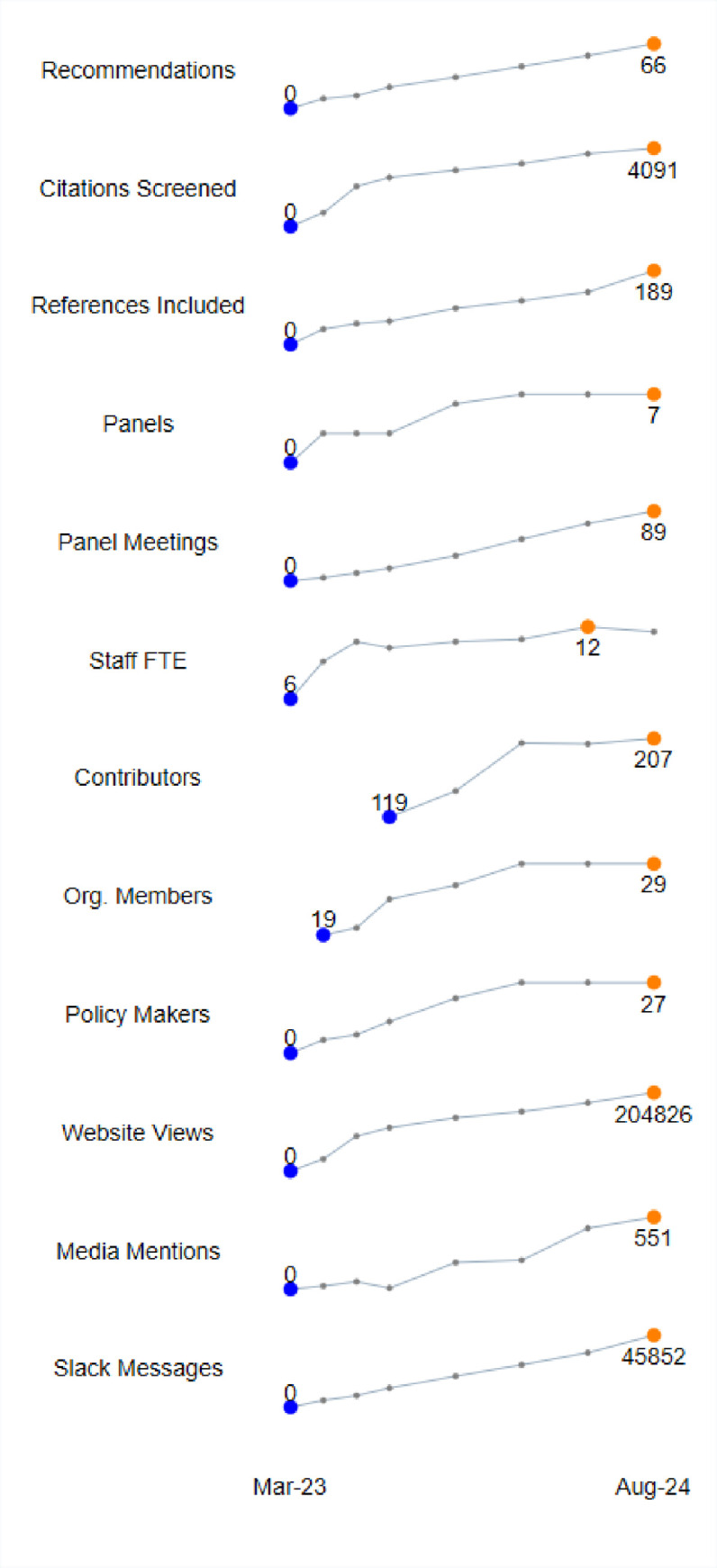
Activity audit data.

Fig 1 provides a summary of the audit data over time. The complete data set is provided in Table 1.

**Table 1:** Activity stocktake.

Item	Unit	Mar-23	Apr-06	Apr-20	May-04	Jun-01	Jun-29	Jul-27	Aug-24
Number of recommendations	Cumulative	0	10	13	22	32	43	54	66
Number of citations screened	Cumulative	0	718	2,098	2,565	2,937	3,284	3,799	4,091
Number of references included	Cumulative	0	40	54	60	93	112	134	189
Number of guideline panels	Count on date	0	3	3	3	6	7	7	7
Number of panel meetings	Cumulative	0	4	10	16	32	53	73	89
Number of staff employed	FTE Count on date	5.7	8.7	10.3	9.8	10.3	10.5	11.5	11.1
Number of individuals engaged in Taskforce	Count on date				119	148	202	201	207
Number of Taskforce members	Count on date		19	20	24	26	29	29	29
Number of Jurisdictional Liaison Group representative members	Count on date	0	5	7	12	21	27	27	27
Number of funders	Count on date				4	5	5	5	5
Number of partners	Count on date				9	8	8	8	8
Number of webpage views	Cumulative	0	31,329	91,506	113,779	139,528	155,573	178,507	204,826
Proportion of webpage views from Australia	% cumulative total	-	87%	88%	86%	83%	80%	78%	76%
Number of media mentions	Count for period	0	24	57	8	203	222	467	551
Number of Slack messages	Cumulative	0	4,343	7,385	12,114	19,915	27,117	34,768	45,852
*Number of Australian COVID-19 cases*	Cumulative	1,681	5,799	6,622	6,825	7,195	7,686	14,403	24,812
*Number of Australian COVID-19 deaths*	Cumulative	7	41	71	95	103	104	155	502
*Number of COVID-19 trials on ClinicalTrials*.*gov*	Cumulative	128	333	692	1,159	1,835	2,346	2,746	3,065

### Quantitative findings from online survey

The first survey was conducted from on May 4–11, seven weeks after the start of the Taskforce. The final survey was conducted from August 24–31, 23 weeks after the start of the Taskforce. The number of people invited to participate in the survey grew from 119 in May, to 207 in August; the response rate declined after the first two rounds, and as the number of individuals contributing to the Taskforce grew. Satisfaction with the work of the Taskforce remained very high (>85% extremely or somewhat satisfied) throughout (see Table 2).

**Table 2:** Survey response rate and levels of satisfaction.

Survey	May-04	Jun-01	Jun-29	Jul-27	Aug-24
Number invited	119	148	202	201	207
Response rate % (n)	38% (45)	32% (48)	14% (29)	15% (30)	16% (34)
**Satisfaction**					
Extremely satisfied	64.5%	56%	55%	70%	65%
Somewhat satisfied	31%	38%	31%	27%	27%
Neutral	4.5%	0%	10%	0%	9%
Somewhat dissatisfied	0%	0%	4%	3%	0%
Extremely dissatisfied	0%	0%	0%	0%	0%
No response	0%	6%	0%	0%	0%

### Qualitative findings from online survey and interviews

A total of 38 interviews were conducted during the process evaluation, five in the first cycle, seven in the second, eight in the third, ten in the fourth and eight in the final cycle. Eight participants were interviewed multiple times (range: 2–5) and the other six participants were interviewed once. Interviewees included 7 members of the Executive Team, 6 members of the evidence synthesis team and a clinical content expert. Data from the interviews were combined with qualitative data collected through the online survey.

#### Strengths

An updated version of the guidelines was successfully published every week during the process evaluation. This consistent delivery of the core output of the Taskforce led to pride and delight in the achievement of the Taskforce. Taskforce contributors were “astonished”, “amazed” and “impressed” by what was achieved and reported that they “feel privileged” to have been involved. “It’s amazing what we’ve achieved.”

Contributors repeatedly emphasised that a core strength of the Taskforce was that the team undertaking the evidence surveillance and living reviews were highly skilled, collegial and committed—“Trust and team work are extraordinary”. The core of the team was drawn from existing Cochrane Australia staff, rapidly bolstered by additional recruitment (often by secondment from within the Australian evidence synthesis community) during the start-up phase. Both having an initial team in place from which to build, and rapid recruitment of experienced individuals to expand this team, were major contributors to success.

In parallel with the expertise in evidence and guideline development methods, respondents regularly highlighted the extremely high levels of collaboration between Taskforce member organisations, and continuing high levels of engagement and contribution from individual clinicians. “The enthusiasm of the individual members is outstanding.”

While the early days of the Taskforce were retrospectively described as “frantic”, clear weekly workflows and processes were quickly established and continually refined. By May, respondents were reporting that the acute establishment phase had passed, and the Taskforce was developing a rhythm and structure; by late July team members reported that operational and evidence processes were running smoothly and efficiently.

#### Challenges

Managing workload for the team was an ongoing challenge. Workloads during the establishment period were extremely high. While this settled a little as workflows were established and team members developed a “much clearer sense of what [they are] here to do”, for some topic areas such as drug treatments this was offset by an increasing rate of study publication. In the last round of the process evaluation there was recognition that “[we] still have people working ‘til midnight”.

The context of the COVID-19 pandemic created additional pressure and stress on the team and other Taskforce contributors, as many faced personal challenges, including increased clinical workloads; and most staff, based in Victoria, Australia, were in lockdown for many months, leading to mental and emotional exhaustion. “[W]e are all in a state of fatigue”. The context also meant it was not feasible for the team to meet face-to-face, and remote working was an additional challenge for communication and efficiency, particularly for new staff without existing relationships with Taskforce colleagues.

The structure of the team presented a challenge as the project progressed. In order to rapidly expand the team, many staff were seconded to the Taskforce for a finite period (most often 3–6 months), and although Taskforce funding was continued beyond this point, some had to return to their existing primary roles, creating skill gaps. Similarly, early expedient decisions to contract external providers for functions such as communication introduced challenges at later stages of the process.

Supporting guideline development panels was an ongoing challenge for the Taskforce. These panels of clinicians each met weekly to consider the evidence provided by the evidence team, and make or update the guideline recommendations. Providing timely, structured, concise but detailed pre- and post-meeting documentation was a significant undertaking. Discussions about the best size for these panels (smaller and more focused/efficient, versus broader and more representative, but less opportunity for discussion), and the scope of the portfolios of work addressed by each panel, continued throughout the period of the process evaluation. Some of the panels functioned effectively throughout, whereas others had periods of inefficiency and lack of clarity about contribution to the Taskforce. Highly skilled clinical chairs and methods co-chairs, were a vital contributor to the success of these panels.

Building on the initial success of the Taskforce, it was proposed the Taskforce expand scope to include infection prevention and control in healthcare settings. Those in favour of expansion saw an opportunity to address issues of vital concern to the guideline audience, however there were also concerns about whether expanding scope would put at risk the delivery of the primary output of the Taskforce, Australian living recommendations for the clinical care of people with COVID-19. These conversations continued throughout the process evaluation, and in the final round views were still mixed: “Exciting opportunities… logical extension”, “a bit scary in terms of resourcing”.

One of the components of the work of the Taskforce is to further build the technology platform to support living guideline production and publication. In the early phases of the work this was felt to be less of an issue, with respondents in June emphasising that “The limit is not tech, it’s humans”. However, the importance of the planned technological improvements was increasingly felt, and by the late August five months into the work, team members were increasingly aware of and frustrated by the tasks that they were doing manually, or things they were unable to do efficiently, because they were beyond the limits of available technology. “[The] tech stuff [is] still really challenging.”

One contributor to the delays in delivery of the technological development was siting of the Taskforce within the Monash University governance structure. There was recognition that Monash’s “admin set-up isn’t as agile as we are”. Location of the Taskforce within a university had important benefits including the provision of core financial and human resources infrastructure and governance oversight enabling rapid establishment, but also introduced significant challenges in the form of additional layers of bureaucracy and delays, especially in contracting. These challenges were exacerbated by the university-wide impacts of the COVID-19 pandemic, both on financial management, and increased administrative workload.

The all-pervasive uncertainty in which the work of the Taskforce was conducted, both in terms of the trajectory and impact of COVID-19 in Australia and globally, and also questions about the future funding and direction of the work of the Taskforce, became less comfortable for contributors over time. In the initial stages, contributors reported that they felt “privileged” to be involved and were altruistically motivated to contribute on an ad hoc basis. By July the Taskforce had reached “a tipping point” that “comes with maturity” where “people’s expectations are different” and there was increasing desire for a clear roadmap that described the future path of the Taskforce and expectations of ongoing contribution. There was also an increased desire for demonstration that the work of the Taskforce was having a meaningful impact. “I don’t have a good sense of whether we are really ‘landing’ with the target audience” “Are people actually using them?”.

#### Methods questions

Two key issues about the living evidence synthesis methods employed in the guidelines were raised repeatedly throughout the process evaluation; the pace of guideline development, and the thresholds for inclusion of evidence.

From the beginning, the Taskforce committed to deliver weekly updates of the guidelines, a pace which was described as “superb”, “relentless” and “onerous”. To meet this aim, a weekly schedule was developed which included meetings of each of the guideline panels, as well as the Guideline Leadership Group to review recommendations, and the Steering Committee to approve recommendations. By June, some respondents were questioning whether weekly meetings were necessary and feasible for all guideline panels, or whether some panels might meet fortnightly or monthly without compromising the value of the guidelines, particularly in areas where less research was being published, or fewer new clinical questions raised. “[The] expectation that every week there will be something new is unrealistic.” Contributors had mixed feelings, with a sense that less frequent meetings were in some way devaluing their contribution, but also frustration if meetings didn’t seem to lead to concrete progress in the form of new recommendations. At the end of the process evaluation this question was still under discussion.

Given the context of a novel disease, with little prior research, initially all evidence was valuable to inform recommendations, and every new study was immediately appraised and incorporated. As the availability of research increased, a question arose about the value of the work required to rapidly incorporate results from small or low-quality studies into evidence summaries when it was clear that these would not impact on current recommendations. Some respondents suggested that an option was needed for a “holding bay”, or that thresholds should be set for incorporation of new evidence, perhaps similar to those used in living systematic reviews. Similarly, methods needed to be developed to deal with increasing numbers of studies available in preprint version.

Many of the key strengths and challenges identified through this process evaluation have application/relevance to other living guidelines (see Box 2). Regular, pre-determined updates are a significant strength of living guideline methodology. During times of acute need, such as a pandemic, weekly updates ensure the most current, evidence-based research is used to inform the rapidly evolving clinical landscape. Engaging highly skilled clinical and methods chairs and evidence team personal ensures efficiency of the panels and quality of the guidelines themselves.

**Box 2. Key strengths and challenges of the living guideline methodology adopted by the Taskforce**

Strengths

*Weekly updates
- Highly skilled clinical chairs, methods co-chairs and evidence team
- Collaboration between member organisations
- High level of engagement of large numbers of clinicians
*Clear, weekly workflows and processes
- University governance–rapid establishment, core human and financial resource infrastructure, governance oversight

Challenges

*Workload management
*Significant external, additional stressors on staff (mental and emotional exhaustion)
- Remote working
*Rapid staff procurement
- Supporting the guideline development panels–ideal size, scope of portfolio of work to be addressed
- Inefficiencies with technology
*University governance–contracting delays
*Uncertainty–trajectory/impact of the pandemic and the guidelines
- Determining appropriate update schedule
*Determining an appropriate, and potentially evolving threshold for inclusion of evidence

*Determining what to do with preprint studies

* Unique strengths/challenges during a pandemic.

Supporting guideline panels is likely to be a consistent challenge across living guidelines however the cumulative effect of supporting numerous members across several panels operating on a weekly update cycle is unique to the experience of the Taskforce. Inefficiencies with technology and determining an appropriate update schedule are challenges likely to be experienced across other living guidelines. Further consideration is required to provide clear recommendations for these areas.

## Discussion

At the conclusion of the process evaluation, as of September 10, the living, evidence-based Australian guidelines for the clinical care of people with COVID-19 had been published and updated 20 times in just over 5 months, and included more than 70 recommendations, a significant achievement for one of the world’s first living guidelines, and certainly the most rapidly updated living guideline to date. Conducting a process evaluation alongside the work of the Taskforce was an important opportunity not only to improve Taskforce processes and outputs as the project progressed, but also to reflect on what future living guidelines and evidence synthesis projects might learn from this experience.

The work of the Taskforce was made possible by the existence of the team at Cochrane Australia with skills in evidence synthesis and guideline development methods, and the Australian Living Evidence Consortium which has been pioneering and piloting methods and platforms for living evidence synthesis [10]. Having this team in place allowed the Taskforce to rapidly ‘spin-up’, providing a core set of skills and leadership which formed the backbone of the team, which was then strengthened over time by recruitment of high-calibre individuals to fill specific skill gaps. This evaluation suggests core skills in evidence synthesis and GRADE guideline development are important determinants of success and would need to be available for other similar projects to progress rapidly.

The second strand of expertise that enabled the Taskforce was convening power. Guideline development relies on the integration of both evidence, consumer and clinical expertise, and the ability to engage and support over 200 contributors and manage their diverse, expert input each week was an important contributor to the credibility of the guidelines. Furthermore, the ability to convene more than 30 organisations representing health practitioners providing care to people with COVID-19 across Australia and to achieve 100% consensus across all these organisations was critical to the position of the guidelines as Australia’s ‘one-stop shop’ for COVID-19 clinical care.

The questions about pace of guideline updating and thresholds for inclusion are important considerations for future living guidelines projects. As in all projects, there is a trade-off between quality, time and resources at the heart of these decisions, and they are also likely to be dependent on the context and the clinical topic under consideration. Decisions about how often new evidence should be considered should be driven by the rate of emergence of new evidence, clinical uncertainty and importance of the area under review. The frequency with which panels are convened does not have to be directly reflected in the publication schedule for the guideline. A guideline with multiple guideline panels like those convened by the Taskforce can, for example, publish weekly updates with only some panels meeting each week. Indeed, this is the model that the Taskforce has now adopted, reflecting the transition from a focus on primarily developing new recommendations for many questions to the less-resource intensive work of maintaining recommendations in living mode.

Existing guidance for living systematic reviews [11] suggests that a priori decisions about thresholds for inclusion of new evidence should be made on the basis of whether the new evidence is likely to change the direction, clinical importance or certainty of the effect. Similar criteria could be useful for decisions about when to include new evidence in evidence summaries for living guideline recommendations, however it will be important to determine how feasible it is to develop these thresholds, how acceptable they are to guideline users and what effect they might have on workload and feasibility. Similarly, there are additional considerations about how to prioritise and select new questions to be addressed by the guidelines over time, and how and when to retire questions from living updates.

The recent rise in publication of living guidelines [2–5] and living systematic reviews [12–22], accelerated by the COVID-19 pandemic, makes addressing these methodological questions increasingly important. This growing body of living evidence syntheses also increases the availability of information about which approaches work best and in which contexts and clinical topic areas, which will be an important contribution to research underway to map these methods [23] and to further development of guidance for living guidelines [1] and other evidence syntheses [11, 24].

This study has some limitations, mostly arising from its pragmatic purpose to inform the conduct of the Taskforce as it was underway. We conducted only a small number of interviews in each round of the process evaluation, however these included all the senior leadership team in each cycle with the exception of two occasions when staff were on leave; along with a selection of other contributors. All Taskforce participants were invited to respond to the survey, and although participation declined over time, numbers were consistently above 25 each cycle, even during school holidays and Melbourne’s second wave of COVID-19. The combination of the regular interviews with the quantitative and qualitative data from the broader survey provides useful insights into the progress and mindset of the Taskforce at each timepoint.

A preliminary analysis of the results of each cycle of the process evaluation were reported to the Taskforce Executive and Steering Committee every four weeks, approximately one week after the data was collected and were used to inform and guide a process of continuous improvement of Taskforce processes and outputs. Providing the results rapidly and contemporaneously meant they could be used iteratively to identify and address emerging issues, and ascertain whether previously raised issues were being effectively addressed, while the project was underway. Some examples of use of the process evaluation data included work to refine roles within the Evidence Team and Executive to improve clarity and workflows; processes to restructure guideline panels to optimise efficiency; creation of deputy chair and methods co-chair roles in each panel to ensure appropriate levels of support; and development of governance mechanisms and standard operating procedures in response to issues raised. The rapid cycle of receipt of feedback through the process evaluation, development of responses by the Taskforce Executive and Steering Committee, and request for further feedback from the next timepoint of the process evaluation, also built confidence that the process evaluation was meaningful, that feedback was being heard, and that contributors’ views were valued. One unexpected benefit of the process evaluation interviews was the opportunity provided to interviewees for a time set aside to pause and reflect, and they felt that the opportunity to step away from the business of their day to day work was helpful both personally and professionally. Living guideline developers should consider establishing a similar model of process evaluation to inform the development and implementation of their guidelines.

Several factors should be considered when interpreting our findings. The most important of these is the context of a global pandemic in which the Taskforce has operated, which has had multiple impacts. The high profile and vital importance of COVID-19 as a global and national health threat was likely a major motivator for the high-levels of engagement in the work of the Taskforce, both for the member organisations and individual clinicians contributing to guideline panels, and for the Taskforce team. It is likely that this, and the resulting urgent demand for recommendations to guide clinical practice, provided an enabling environment for the high levels of collaboration and cooperation seen in the Taskforce. The COVID-19 context also placed additional pressure and stress on the team and Taskforce contributors, as many faced significant additional personal and professional challenges, including an extended lockdown period for staff based in Victoria. Remote working was the norm for the Taskforce, with staff working from home—every team, panel and group meeting was conducted in an online environment. These contextual factors perhaps limit generalisability of some of our findings, but the global pandemic also provides a useful stress-test of the living evidence model, demonstrating that it is possible to develop rigorous, evidence-based living guidelines even during times of crisis. This process evaluation presents useful learnings on the rapid development and establishment of living guidelines during a pandemic. We have demonstrated that rapid development of living GRADE-based guidelines is both feasible and acceptable and provided some insights to the strengths and challenges of living guideline methods in general.

## Conclusion

The Australian Guidelines for the Clinical Care of People with COVID-19 are an important example of intensive application of living guideline methods. Despite the challenges of implementing a novel approach to evidence-based guideline development remotely during a pandemic, the Taskforce has demonstrated the feasibility of very frequent updating for topics in which stakeholders value very high levels of guideline currency.

The National COVID-19 Clinical Evidence Taskforce process evaluation provided useful data to improve the guidelines and the processes by which they were produced; and identified important considerations for future living guideline projects. An impact assessment is also being conducted to examine the extent of awareness, acceptance and use of the guidelines.
